# Quality of Life and Mental Health in COVID-ARDS Survivors After V-V ECMO Support: Results from the Freiburg ECMO Outcome Study (FEOS)

**DOI:** 10.3390/jcm14072206

**Published:** 2025-03-24

**Authors:** Dawid L. Staudacher, Meret Felder, Markus Jäckel, Felix A. Rottmann, Alexander Supady, Xavier Bemtgen, Philipp Diehl, Tobias Wengenmayer, Viviane Zotzmann

**Affiliations:** 1 Interdisciplinary Medical Intensive Care, Faculty of Medicine and Medical Center, University of Freiburg, 79085 Freiburg, Germany; dawid.staudacher@uniklinik-freiburg.de (D.L.S.); meret.felder@gmail.com (M.F.); alexander.supady@uniklinik-freiburg.de (A.S.); xavier.bemtgen@uniklinik-freiburg.de (X.B.); tobias.wengenmayer@uniklinik-freiburg.de (T.W.); 2 Department of Cardiology and Angiology, Faculty of Medicine, Heart Center Freiburg University, University of Freiburg, 79085 Freiburg, Germany; 3 Department of Medicine IV, Nephrology and Primary Care, Faculty of Medicine and Medical Center, University of Freiburg, 79085 Freiburg, Germany; felix.rottmann@uniklinik-freiburg.de; 4 Department of Cardiology, Pneumology, Angiology, Geriatrics, Intensive Care Medicine and Thoracic Surgery, Ortenau Clinical Center Offenburg, 77654 Offenburg, Germany; philipp.diehl@ortenau-klinikum.de

**Keywords:** acute respiratory distress syndrome (ARDS), venovenous extracorporeal membrane oxygenation (V-V ECMO), COVID-19 (coronavirus disease 2019), long-term survival, health-related quality of life (HRQoL), SF-36 health survey (short form-36)

## Abstract

**Introduction**: Desirable outcome after venovenous extracorporeal membrane oxygenation for acute respiratory distress syndrome (ARDS) is frequently defined by survival. However, quality of life (QoL) and mental health status may take precedence over mere survival, from a patient-centered perspective. We aimed to evaluate QoL and mental health status in survivors after V-V ECMO for coronavirus disease 2019 (COVID-19)-related ARDS, hypothesizing a similar QoL comparable to the general population. **Methods**: All patients supported with venovenous ECMO for COVID-19-related ARDS between 01/2020 and 03/2022 in our center were included. Survivors were invited to participate in a follow-up interview assessing QoL, anxiety, and depression one year after hospital discharge. Primary endpoint was the quality of life, measured by the SF-36 questionnaire, with results compared to data from the DEGS1 study (German normative population). **Results**: During the study period, 97 patients received venovenous ECMO for COVID-19 ARDS at our ICU. Overall, 43/97 (44.3%) survived, and 21/97 (21.6%) completed the SF-36 questionnaire. The median follow-up duration was 1.7 years. Patients who completed the SF-36 were significantly younger than those who did not (48.7 vs. 55.6 years, *p* = 0.012); other patient characteristics and ECMO parameters were similar between those with and without questionnaire. Anxiety, depression, and post-traumatic stress disorder were detected in 33%, 14%, and 29% of patients, respectively. Compared to the German normative population, ECMO survivors had significantly lower QoL (mean 77.2 vs. 61.0, *p* < 0.001). **Conclusions**: QoL and mental health status after venovenous ECMO for ARDS was significantly lower compared to the normative population. These findings highlight the importance of further research and comprehensive follow-up care for ECMO survivors.

## 1. Introduction

Severe acute viral infections of the respiratory tract may lead to acute respiratory distress syndrome (ARDS) associated with high in-hospital mortality. Initial case reports and case series on the use of venovenous ECMO (V-V ECMO) for coronavirus disease 2019 (COVID-19)-related ARDS were disappointing and raised concerns regarding the use of extracorporeal membrane oxygenation (ECMO) in this patient population [1,2]. However, more recent data from large cohorts of COVID-19 ARDS patients supported with ECMO have revealed considerably better outcomes, similar to those after ARDS not related to COVID-19. During the height of the COVID-19 pandemic, V-V ECMO played a pivotal role in managing patients with very severe courses of the disease [3,4,5].

The long-term consequences of such severe diseases even impact patients after recovery of the acute disease state [6,7,8].

The consequences of prolonged intensive care therapy, particularly for patients subjected to long-term mechanical ventilation, are manifold and often extend beyond the immediate recovery phase. Long-term mechanical ventilation, which may become necessary after severe ARDS, can profoundly impact both the physiological and psychological aspects of a patient’s well-being [9].

Physiological consequences include muscle weakness and atrophy due to prolonged immobilization and inactivity, which are known to be particularly pronounced in the major muscle groups, affecting mobility and overall physical function [10]. Additionally, extended mechanical ventilation periods increase the risk of complications such as ventilator-associated pneumonia (VAP) and ventilation-induced lung injury (VILI). VAP can further compromise respiratory function, leading to extended patient recovery. States of critical illness are often associated with organ dysfunctions including the cardiopulmonary system, the kidneys, and the liver, with consecutive long-term health issues.

Psychological consequences: the combination of physical and psychological effects after intensive care treatment are known to significantly impact patients’ quality of life and often persist long after the acute phase of disease [11]. Survivors of long-term intensive care unit (ICU) stays, especially those with extended periods of mechanical ventilation, are at increased risk for developing post-traumatic stress disorder (PTSD) due to lasting psychological scars [10,12]. Depression and anxiety are frequently reported after prolonged intensive care stays. Feelings of helplessness, uncertainty about the future, and the trauma associated with critical illness routinely impact mental health [10,12]. Furthermore, impaired cognitive ability appears frequently in those patients, resulting in cognitive disorders and the inability to resume normal daily activities [13].

The combination of physical, cognitive, and psychological issues that persist after ICU discharge is often referred to as post-ICU syndrome (PICS). However, there is only little evidence about parameters causing PICS. Investigating and addressing PICS is crucial for developing comprehensive post-intensive care rehabilitation strategies, thereby improving the quality of life of our patients.

The Freiburg COVID-ECMO outcome study (FEOS) aims to delve into the post-treatment landscape, specifically focusing on the quality of life experienced by individuals who underwent V-V ECMO for COVID-19 ARDS. Understanding and evaluating the post-ARDS life experience involves a multidimensional approach. This paper concentrates on employing three well-established measurement tools: the Short Form-36 health survey (SF-36) [14,15], the Hospital Anxiety and Depression Scale (HADS) [16,17], and the Impact of Event Scale-Revised (IES-R) [18,19]. These instruments collectively provide a comprehensive assessment of various aspects of an individual’s well-being, covering physical and mental health as well as the psychological impact of traumatic events.

The hypothesis in FEOS is that, once the acute COVID-19 ARDS was resolved, patients surviving at least a year would have a good quality of life comparable to a German reference cohort. Primary endpoint was the quality of life, measured by the SF-36 questionnaire.

## 2. Materials and Methods

### 2.1. Freiburg COVID-ECMO Outcome Study (FEOS)

Patient characteristics were derived from a single-center registry. All quality-of-life assessments were conducted prospectively. The study was approved by the Ethics Committee of the Albert Ludwig University of Freiburg (ethic application no 21-1254) and supported by a grant from the European Chapter of the Extracorporeal Life Support Organization (EuroELSO). The follow-up was carried out at the University Hospital of Freiburg, Germany.

### 2.2. Patient Cohort

All patients treated for COVID-19 ARDS and supported with V-V ECMO were included (all-comer study). The following inclusion criteria were applied in FEOS: (1) SARS-CoV-2 detection by PCR or antibody test, (2) COVID-19 ARDS main diagnosis for ICU treatment, (3) V-V ECMO support for COVID-19 ARDS, and (4) age 18 years or above at ECMO cannulation. The following exclusion criterion was applied: (1) presentation after May 2022. All patients were therefore admitted between 30 March 2020 and 31 March 2022. All ICU survivors were contacted by mail between October and November 2022. The mailing included a cover letter providing information about the background, goals, and process of the research project. Patients were invited to participate in a follow-up examination and quality-of-life questionnaires. The mailing also included a declaration of consent or refusal and a stamped return envelope. After receiving written informed consent, appointments for the follow-up were scheduled between November 2022 and February 2023.

### 2.3. Group Formation

The first part of this research compares patients with and without completed follow-up questionnaires. The non-follow-up group therefore compromised patients dying during the initial ICU course or after discharge, those refusing to participate in the study, and those lost to follow-up. In those with complete follow-up, we predefined to compare patients according to biological sex, based on available records, as gender was not directly assessed.

### 2.4. Treating Institution

The Freiburg University Hospital is a tertiary care center and served as a primary referral hub during the COVID-19 pandemic. Indication for V-V ECMO was based on the EOLIA criteria [20]. Cannulation was performed both in referral clinics as well as in our hospital and both dual-lumen and single-lumen cannulas were used. ARDS therapy was administered according to the guidelines for ARDS and COVID-19 that were current at the time.

### 2.5. Health-Related Quality-of-Life (HRQoL) Assessment

Three different questionnaires were sent to the patients in advance as a document or, in the case of the SF-36, as a link by email. The questionnaires could either be processed in advance and then returned by email or brought by the patient to the appointment. If not processed before the time of the follow-up examination, a printed version could be processed on site together with one of our researchers (MF) in order to help with language and to ensure data quality. Questionnaires were tested in this order: (1) SF-36 if not prefilled, (2) HADS, (3) IES-R, and (4) a tailored questionnaire covering personal and demographic data as well as on the ICU stay.

The Short Form-36 questionnaire is a widely used health-related quality-of-life questionnaire designed to assess an individual’s overall well-being across various physical and mental health domains [11]. It was developed by the Medical Outcomes Study (MOS) and has become one of the most commonly employed instruments for measuring health status [14,15]. The SF-36 consists of 36 questions that cover eight health concepts or domains. These domains are further divided into two main categories: physical health and mental health. Participants answered questions based on their experiences over the past four weeks, and each domain is scored separately. Higher scores indicate better health and functioning. Reference values for the German population include the Federal Health Survey 1998 [15] and the results of the study on the health of adults in Germany (DEGS1) from 2013 [14] were used. The questionnaires are evaluated computer-aided using the online program provided. The results of the respective items in the individual dimensions are summed up and then transformed into a scale from 0 to 100. Higher values reflect a better health-related quality of life in this area [14]. The processing and transmission of the SF-36 results took place online using an individually generated link, provided by Hofgrefe Publishing in a German version [20].

### 2.6. Hospital Anxiety and Depression Scale (HADS) Questionnaire

The HADS is a self-report questionnaire designed to assess the levels of anxiety and depression in individuals who are patients in a hospital setting. It was developed by Zigmond and Snaith in 1983 [16]. The HADS is widely used in medical and psychological research, as well as in clinical settings in post-ECMO survivors [21], to screen for and measure the severity of anxiety and depression symptoms. The scale is often used to identify individuals at risk of anxiety and depression in a hospital or healthcare setting. The HADS consists of fourteen items, with seven items dedicated to measuring anxiety and seven items for depression. Participants are asked to rate the extent to which they have experienced specific symptoms over the past week. Each item is scored on a Likert scale, typically ranging from 0 to 3, with higher scores indicating higher levels of anxiety or depression conditions [17]. While the HADS is a valuable screening tool, it is important to note that it is not a diagnostic tool. Elevated scores on the HADS may suggest the presence of anxiety or depression, but a comprehensive clinical assessment is typically needed to confirm a diagnosis and determine appropriate interventions. We used the 4th updated German version (2018) of the questionnaire from Herrmann-Lingen C [22].

### 2.7. Impact of Event Scale-Revised (IES-R) Questionnaire

IES-R is a psychological instrument designed to measure the impact of stressful life events and their effects on an individual’s emotional well-being [18]. The IES-R test is commonly used in clinical psychology and psychological research to assess the extent of post-traumatic stress in individuals who have experienced traumatic events [23]. The IES-R test focuses specifically on assessing post-traumatic stress. Participants needed to answer 22 questions and were asked to indicate the extent to which specific statements apply to them, based on their experiences with a particular stressful event. The total score provides insight into the degree to which an individual is affected by the impact of trauma. The test comprises 22 items divided into three subscales: intrusion, avoidance, and hyperarousal. These subscales capture various aspects of post-traumatic stress [18,22]. The intrusion scale measures the unwanted re-experiencing of distressing memories, the avoidance scale assesses strategies used to avoid traumatic events, and the hyperarousal scale measures heightened arousal and anxiety. The suspected diagnosis of PTB is calculated according to the following formula: X = (−0.02 (×) intrusion) + (0.07 (×) avoidance) + (0.15 (×) hyperarousal) − 4.36 [19].

### 2.8. Additional Questionnaire

A tailored questionnaire on social status, employment before and after the inpatient stay, and questions about the experience of the ICU itself was presented.

### 2.9. Statistical Methods

Continuous variables are given as mean (minimum—maximum), while categorical variables are given as number in that category (percentage of group). Significance is calculated between groups using the Mann Whitney test (since a normal distribution could not be presumed in all cases) or the Fisher’s exact test as applicable. Series of continuous variables as for the SF-36 were compared using a 2-way ANOVA. *p*-values below 0.05 were considered statistically significant. For comparison of the SF-36 values of the German cohort, ANOVA was employed, which necessitated transformation of the data to mean and standard deviation from the confidence intervals given in the publication. Therefore, we calculated the margin of error as half the width of the confidence interval. Using the margin of error and the Z-value corresponding to the 95% confidence level, we then determined the standard error. Finally, the standard deviation was obtained by multiplying the standard error by the square root of the sample size. Data were organized using Microsoft Excel (Microsoft, USA) and significances were calculated using Prism 10.4.1 (GraphPad, La Jolla, CA, USA) or SPSS Statistics 27 (IBM, Armonk, NY, USA).

## 3. Results

**Figure 1:**
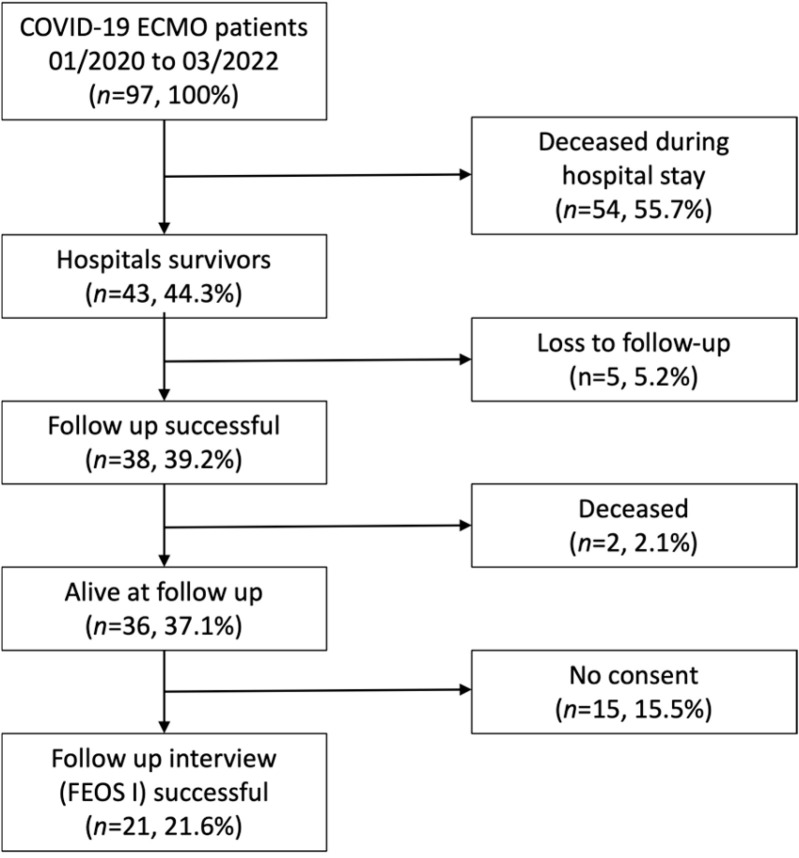
Study flow chart of recruitment.

Patient cohort: FEOS included 97 patients with COVID-19 ARDS undergoing V-V ECMO between 1 March 2020 and 31 March 2020. Of these, 41/97 (44.3%) survived until discharge (hospital survival). Of the 41 hospital survivors, 5 were lost to follow-up and 15 denied participation in this study, resulting in 21/97 (21.6%) patients with quality-of-life assessment (see Figure 1). Two patients died early (during the first 6 months) after discharge. The mean follow-up period between V-V ECMO implantation and follow-up examination was 20.3 ± 7.2 months. Long-term survival (as assessed between November 2022 and February 2023) was at least 37.1% (*n* = 36/97).

Comparing patients with QoL assessment (the follow-up group) to those without follow-up (the no-follow-up group), patients in the follow-up group were significantly younger at the time of V-V ECMO cannulation (49 versus 56 years, respectively, *p* = 0.012). When only considering hospital survivors with and without follow-up, age was similar (49 versus 52 (18–68) years, *p* = 0.5535). All other patient characteristics, including sex, body mass index, all investigated comorbidities, and the Charlson comorbidity scale, were similar (all *p* > 0.10); see Table 1.

At the time of ECMO cannulation, all investigated blood gas samples (including arterial pO_2_, pCO_2_, and pH) were similar in patients with and without follow-up. Concerning parameters of mechanical ventilation, the Horovitz index (pO_2_ divided by FiO_2_) was significantly higher in patients in the follow-up group (85 versus 75 mmHg, respectively, *p* = 0.033). All other ventilator settings including driving pressure, tidal volume, and compliance were similar; see Table 2.

Comparing treatments, the durations of ICU stay, hospital stay, mechanical ventilation, and ECMO were similar in patients with and without follow-up. Outcome, however, was significantly better in patients with follow-up, most notably the better long-term survival rate in patients with follow-up (100 versus 26%, respectively, *p* < 0.001); see Table 2.

**Figure 2:**
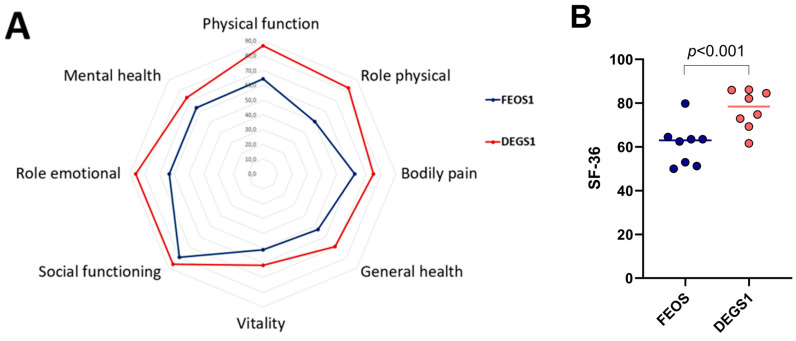
Health-related quality of life as estimated by the SF-36 score in FEOS (long-term follow-up of COVID-V-V ECMO survivors, blue color) and DEGS1 (German reference cohort, red color). Higher scores denote better health-related quality of life. (**A**): bullseye plot. (**B**): dot plot of both scores.

**Figure 3:**
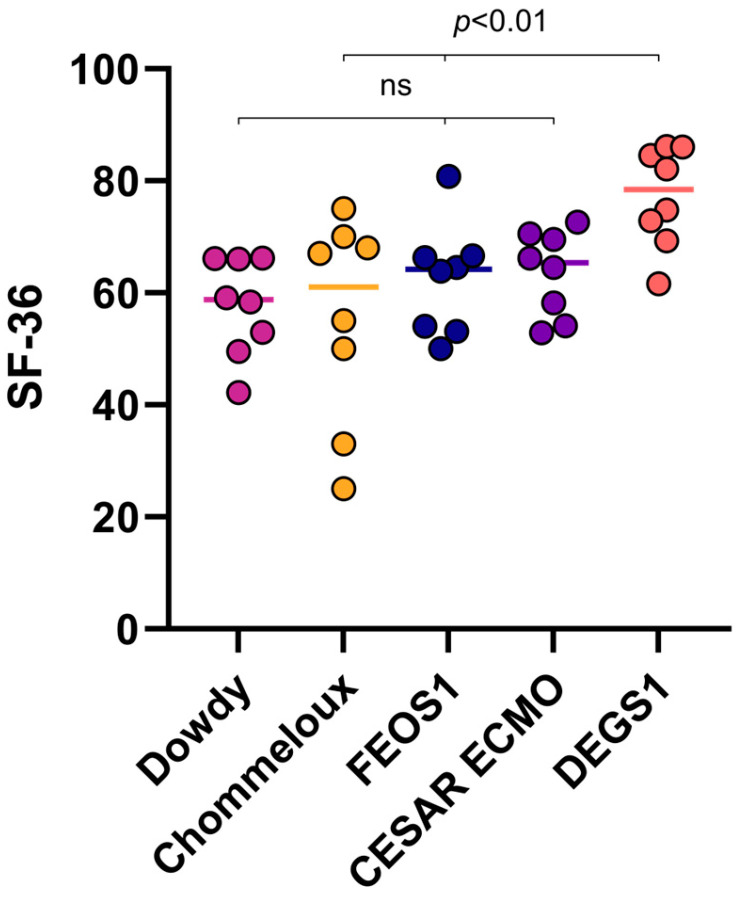
Comparison of different sudies or meta-analyses of health-related quality of life using the SF-36 form in different patient collectives compared to FEOS (Freiburg COVID-ECMO outcome study). Included studies are Dowdy et al. [23], Chommeloux et al. [24] Peek et al. [25], Ellert et al. [14].

A total of 21 patients completed the SF-36 questionnaires. The SF-36 was filled out autonomously in 20/21 patients. In one case, the patient had difficulty opening the link, which required a procession of the SF-36 by phone. There was no difference between male and female patients concerning the SF-36 form. Comparing the SF-36 in our patient group to a German norm group evaluated in the DEGS1 study, we found significantly lower quality of life in our patient group (62.4 versus 77.2, respectively, *p* < 0.001); see Figure 2. Of the eight items compared in the SF-36 questionnaire, only four were significantly lower. Bodily pain, vitality, social role functioning, and mental health were similar between both groups; see Supplementary Table S1. Figure 3 shows a comparison of different sudies or meta-analyses of health-related quality of life using the SF-36 form in different patient collectives compared to FEOS.

Anxiety and depression were assessed using the HADS questionnaire, identifying HADS anxiety in 33% and HADS depression in 14% of all patients. All patients who showed abnormalities in the HADS depression score also showed signs of an anxiety disorder. HADS depression was pathological in three participants, two of them female; see Table 3. Risk for a post-traumatic stress disorder was found in 6/21 (29%) patients showing an IES-R > 0. Among these patients, the rate of affected women was numerically higher compared to the rate of men (50% versus 15%, respectively, *p* = 0.146); see Table 3.

**Figure 4:**
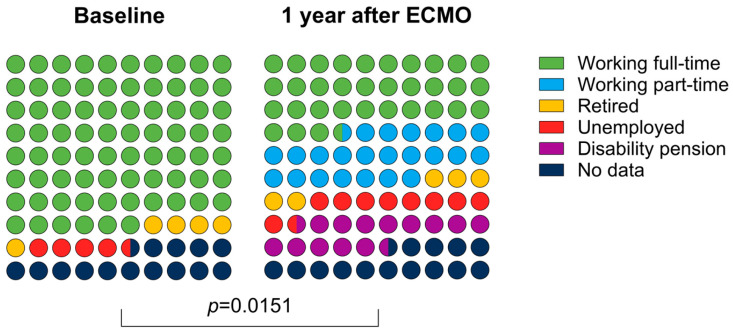
Distribution of patients who were able to return to work (full-time, part-time) after discharge, were retired, unemployed, or were no longer able to work.

The working status changed significantly one year after ECMO compared to the status before the ARDS. Still, 12/21 (57%) could return to work either full- or part-time; see Figure 4.

## 4. Discussion

We examined the quality of life and the occurrence of anxiety disorders, depression, and post-traumatic stress disorder after prolonged intensive care stay in critically ill COVID-19 ARDS patients who were supported with V-V ECMO.

### 4.1. Quality of Life

Comparing the SF-36 score of the patients followed-up after ECMO to a German norm population [14], we found a significantly lower quality of life in ECMO survivors included in FEOS. Looking at the individual subcategories of the SF-36 score, four were similar between FEOS and the German norm population. Physical quality of life was reduced more pronouncedly compared to mental items. Comparing our data to other studies investigating quality of life after ECMO, we found a similar quality as reported by the CESAR ECMO study [25] and significantly lower quality of life than reported by Schmidt et al. [26] and Rilinger et al. [21]. Quality of life found in FEOS, however, is comparable to the quality of life reported in patients with ARDS without ECMO. A meta-analysis of ARDS survivors reported similar findings of lower physical than mental quality of life and on average 15 lower than population norms [23]. We can only speculate on why the quality of life reported in other post-ECMO studies seemed to be better than in our current analysis. Although we found only few significant differences in data derived from the hospital stay between patients lost to follow-up and those included, we cannot rule out a selection bias with patients with lower quality of life agreeing to our interview. However, what is striking is that the average age in the other studies is at least 10 years younger. In the PRESERVE trial it is 44 years [26], and in the CESAR trial it is 39.9 years [25], while in FEOS 1 it is 54.2 years.

Also, COVID-19 might have played a role. Follow-up examinations without ECMO, both in patients with SARS-CoV2 pneumonia [27] and with COVID-19 ARDS requiring intensive care [28,29], showed significantly reduced values in the mental and physical score of the SF-36 questionnaire. This could underline the consequences of COVID as one reason for the poorer SF-36 values.

Still, our data are worrisome and more data on quality of life after ECMO are necessary since we cannot be sure that quality of life after ECMO is as good as it is in the population norm.

Notably, more than half of the ECMO survivors could return to work at least part-time. This rate is comparable with the results from the PRESERVE trial (52%) [26] as well as the study from Herridge et al. [30] (48% after one year, 65% after two years) in patients treated for ARDS without ECMO. The rate is even higher than in a French study (38%) that also examined the quality of life after V-V ECMO in COVID patients [24].

### 4.2. Anxiety and Depression

We found evidence for anxiety and depression in 33% and 14%, respectively. Comparing to the German general population, incidence of anxiety and depression are reported in 14% and 10%, respectively [31,32]. A similar rate was reported in other European countries [33,34]. It is known that the prevalence of depression and anxiety in ICU survivors is high [35]. A national multicenter study in the USA [36] investigated the frequency of psychiatric symptoms by evaluating the HADS in 1-year-ARDS survivors. A total of 416 of 629 patients (66%) were identified with substantial symptoms in at least one domain. A recent review found a prevalence of depression and anxiety among ICU survivors of over 20% [37]. Similar results were reported by a multi-center trial reporting depression after ARDS in 26% [38]. Only few studies report rates above 60% [36].

Looking at V-V ECMO patients, Lin et al. found depression in 28% [39], while a French study reported depression and anxiety in 44% and 42%, respectively.

Focusing on COVID patients with ARDS, the rate of depression 26.3% [40], 36%, and anxiety 34% [29] seems to be in the same range.

Anxiety and depression found in FEOS, therefore, are well in the range of the literature of ARDS (with and without COVID, with and without ECMO). We found no differences between men and women in FEOS 1. This applies to both the rate of depression and anxiety (see Table 3).

### 4.3. Post-Traumatic Stress

We found evidence for PTSD in 29% of patients.

Patients who were not treated in intensive care show a lower prevalence of PTSD of approximately 12% [41,42]. However, it is well known that ICU survivors are susceptible to PTSD. A large meta-analysis from 2015 [43], which included *n* = 4260 patients, examined the occurrence of PTSD within 6 months or 12 months after ICU stay. In most of the included studies, the IES test was also the most common post-traumatic disorder instrument. The range for PTSD in all studies showed a wide range between 4 and 62%, and the pooled prevalence of PTSD was 24% within 6 months after ICU discharge, or 22% within 12 months. Another systematic review [23] also showed very similar results, with a pooled prevalence of 22% in ICU survivors.

When comparing V-V ECMO patients with regard to PTSD, inconsistent results are shown in the literature.

Both Schmidt et al. (PRESERVE) [26] and a study from the Karolinska Institute in Stockholm [43] showed low prevalence of PTSD in ARDS patients treated with V-V ECMO (16% and 14%, respectively). On the other hand, Sanfilippo et al. [44] showed a noticeable higher prevalence of PTSD with 42%.

The same applies to COVID-ARDS patients who were supported by V-V ECMO. Here, too, the results of previous studies are inconsistent. While a large Spanish study [29] and Chommeloux et al. [24] reported a high prevalence of moderate-to-severe PTSD at 49.5%, Guenther et al. [40] found PTSD in only 15.8% of patients.

In summary, a range of PTSD incidence of 14–42% can be observed for ARDS and COVID-ARDS patients supported by V-V ECMO. The prevalence of PTSD in FEOS, therefore, is well in the range reported by the literature.

Considering risk factors for the occurrence of PTSD, two large reviews [37,45] were able to identify the use of benzodiazepines and the occurrence of flashbacks to frightening situations during the ICU stay as risk factors. Female gender and young age are also often associated with PTSD [45,46], although the data on this are not consistent. What is always evident, however, is that male gender and older age have never appeared as a risk factor for PTSD.

What is remarkable in FEOS 1 is that two-thirds of the PTSD patients were women. The age of those with evidence of PTSD was also significantly younger compared to those without PTSD (*p* < 0.05, see Supplementary Table S2). Due to the very small number of cases, this cannot be used further, but it fits with the reviews mentioned above.

We were not able to demonstrate an association with the use of benzodiazepines.

What was also remarkable in FEOS 1 was that the patients with signs of PTSD had significantly lower arterial pO_2_ values at the time when connected to V-V ECMO than the patients who did not develop PTSD (*p* < 0.05, see Supplementary Table S2). There is no direct comparative data for this in the literature. However, there is evidence that low saturation affects quality of life. This was shown by results from the DACAPO study by Bein et al. [47,48], which reported that desaturations with SaO_2_ <85% were reflected in a significant deterioration in QoL in the mental score (SF12).

### 4.4. Limitation

This is a retrospective analysis of a heterogeneous patient cohort, with a significant portion of the initial cohort lost to follow-up. Several potential biases need to be considered, including selection bias, confounding bias, information bias, recall bias, and reporting bias. The recall bias might also have significantly lowered the reporting of any psychiatric symptoms. Results should be interpreted cautiously and considered hypothesis-generating only. Due to study limitations, these findings cannot be assumed to apply to all post-ECMO survivors.

## 5. Conclusions

Quality of life of V-V ECMO survivors was significantly lower compared to the population norm. In particular, the physical role was compromised after ECMO. Post-traumatic stress, depression and anxiety were high.

These findings underline the need for further research to identify risk factors in order to better address them during therapy, as well as the need for comprehensive follow-up care after ECMO support to improve quality of life and mental health. Given the importance of quality of life in patient care, we propose that ECMO centers be required to perform regular follow-ups and report outcomes, with registries actively supporting these measures.

## Figures and Tables

**Table 1:** Baseline patient characteristics. Given are the whole cohort and patients divided into those with and without follow-up. Data are given as mean (min–max) or *n* (percentage of group) as applicable. Significance is calculated between patients separated by the follow-up status and either the Mann Whitney test or Fisher’s exact test was used as applicable. *p*-values below the significance threshold are given in bold. Abbreviations: BMI (body mass index), ECMO (extracorporeal membrane oxygenation), AMI (acute myocardial infarction).

Patient Characteristics	Whole Cohort	Follow-Up Group	No-Follow-Up Group	*p*-Value
*n*=	97 (100%)	21 (21.6%)	76 (78.4%)	
Follow-up available	21 (21.6%)	21 (100%)	0 (0%)	
Age at ECMO cannulation	54.2 (18–74)	48.7 (28–66)	55.6 (18–74)	**0.0121**
Male sex	65 (67.0%)	13 (61.9%)	52 (68.4%)	0.6062
Height	173.2 (155–190)	175.2 (156–190)	172.2 (155–190)	0.0947
Weight	95.7 (60–185)	97.7 (70–150)	95.1 (60–185)	0.3956
Body mass index (BMI)	32.1 (20.9–58.8)	32.2 (21.0–47.4)	32.1 (20.9–58.8)	0.8800
Charlson comorbidity scale	0.8 (0–9)	0.5 (0–3)	1.0 (0–9)	0.1071
Comorbidities				
Chronic heart failure	4 (4.1%)	0 (0%)	4 (5.5%)	0.2830
Chronic pulmonary failure	15 (15.5%)	4 (19.0%)	11 (14.5%)	0.6079
Chronic liver failure	3 (3.1%)	1 (4.7%)	2 (2.6%)	0.6177
Chronic kidney failure	0 (0%)	0 (0%)	0 (0%)	>0.9999
Previous AMI	9 (9.3%)	0 (0%)	9 (11.8%)	0.0978
Immunosuppression	4 (4.2%)	1 (4.7%)	3 (3.9%)	0.8680
Cerebral arteriosclerosis	1 (1.0%)	0 (0%)	1 (1.3%)	0.5972
Dementia	1 (1.0%)	0 (0%)	1 (1.3%)	0.5972
Diabetes mellitus	20 (20.6%)	3 (14.3%)	17 (22.4%)	0.4177
Malignant disease	8 (8.2%)	0 (0%)	8 (10.5%)	0.1206

**Table 2:** Clinical course and outcome. Given are characteristics at the time of ECMO cannulations and the outcome of the whole cohort and of patients divided into those with and without follow-up available. Data are given as mean (min–max) or *n* (percentage of group) as applicable. Significance is calculated between patients separated by the follow-up status and either the Mann Whitney test or Fisher’s exact test was used as applicable. *p*-values below the significance threshold are given in bold. Abbreviations: ECMO (extracorporeal membrane oxygenation), ICU (intensive care unit), IMV (invasive mechanical ventilation), MV (mechanical ventilation), PEEP (positive end-expiratory pressure), POCT (point-of-care testing).

Clinical Course and Outcome	Whole Cohort	Follow-Up Group	No-Follow-Up Group	*p*-Value
*n*=	97 (100%)	21 (21.6%)	76 (78.4%)	
Follow-up available	21 (21.6%)	21 (100%)	0 (0%)	
Follow-up after [years]	1.7 (1.1–2.7)	1.7 (1.1–2.7)	0	
POCT at ECMO cannulation				
pCO_2_ [mmHg]	64.2 (29–150)	64.5 (34–150)	64.2 (29–138)	0.7229
pH []	7.27 (6.92–7.49)	7.27 (6.94–7.47)	7.27 (6.92–7.49)	0.9186
paO_2_ [mmHg]	64.6 (33–138)	59.6 (39–89)	66.1 (33–138)	0.1283
Lactate [mmol/L]	2.3 (0.3–21.0)	1.6 (0.6–2.8)	2.4 (0.3–21.0)	0.4885
SaO_2_ [%]	86.4 (36–99)	85.3 (73–94)	86.7 (36–99)	0.1283
MV at ECMO cannulation				
PEEP [cmH_2_O]	14.9 (5–38)	14.2 (5–18)	15.0 (9–38)	0.6562
Ppeak [cmH_2_O]	32.7 (15–46)	31.5 (23–45)	33.0 (15–46)	0.1205
Horovitz [mmHg]	77.5 (33–265)	84.9 (39–114)	75.4 (33–265)	**0.0332**
Tidal volume [mL]	404.9 (18–1067)	422.1 (62.2–888)	400.3 (18–1067)	0.4475
Compliance [ml/cmH_2_O]	28.5 (3.73–138)	32.3 (3.73–138)	27.5 (8.31–98)	0.4153
Hospital stay [days]	47.1 (1–146)	60.3 (17–134)	43.3 (1–146)	**0.0074**
ICU stay [days]	43.7 (1–143)	51.3 (16–109)	41.5 (1–143)	0.0546
IMV duration [days]	40.6 (0.4–143)	47.1 (11–100)	38.8 (0.4–143)	0.0724
ECMO duration [days]	25.8 (0.2–135.5)	29.1 (2.5–79)	24.9 (0.2–135.5)	0.2043
Deceased in ICU	54 (55.7%)	0 (0%)	54 (71.0%)	**<0.0001**
Modified ranking score	5.2 (2–6)	2.9 (2–5)	5.6 (3–6)	**<0.0001**
Long-term survival	41 (42.3%)	21 (100%)	20 (26.3%)	**<0.0001**

**Table 3:** Functional outcome. Given are quality of life and functional outcome after V-V ECMO survival. Data are divided into the whole cohort and stratified by sex. Data are given as mean (min–max) or *n* (percentage of group) as applicable. Significance is calculated between male and female patients using the Mann Whitney test or Fisher’s exact test. Abbreviations: HADS: Hospital Anxiety and Depression Scale, IES-R: Impact of Event Scale—Revised, PTSD: Post-Traumatic Stress Disorder, SF-36: Short Form-36 Health Survey.

Functional Outcome	All	Female	Male	Significance
*n*=	21 (100%)	8 (38.1%)	13 (61.9%)	
**Quality of Life (SF-36)**				
Physical functioning	64.5 (15–100)	61.3 (20–85)	66.5 (15–100)	0.5808
Physical role functioning	50.0 (0–100)	50.0 (0–100)	50.0 (0–100)	0.9033
Bodily pain	63.9 (22–100)	63.1 (22–100)	64.3 (22–100)	0.9329
General health perceptions	54.0 (0–77)	52.8 (0–72)	54.7 (25–77)	0.9006
Vitality	53.1 (5–90)	49.4 (5–70)	55.4 (30–90)	0.7642
Social role functioning	80.8 (12.5–100)	88.8 (60–100)	76.0 (12.5–100)	0.3368
Emotional role functioning	66.6 (0–100)	66.7 (0–100)	66.6 (0–100)	0.9932
Mental health	66.3 (16–96)	60.5 (16–92)	69.8 (36–96)	0.3618
Change in health status	1.8 (1–5)	1.3 (1–2)	2.1 (1–5)	0.0605
Physical sum score	41.0 (25.2–57.7)	40.8 (25.2–48.2)	41.2 (27.8–57.7)	0.8738
Mental sum score	48.6 (29.3–60.1)	47.7 (34.8–59.6)	49.1 (29.3–60.1)	0.4886
**Hospital Anxiety and Depression (HADS)**				
Anxiety	6.8 (0–15)	7.8 (2–15)	6.2 (0–13)	0.3818
Inconspicuous	14 (66.6%)	6 (75.0%)	8 (61.5%)	0.6557
Suspicion of/secured	7 (33.3%)	2 (25.0%)	5 (38.5%)	0.6557
Depression	4.1 (0–14)	4.6 (1–14)	3.8 (0–10)	0.8433
Inconspicuous	18 (85.7%)	6 (75.0%)	12 (92.3%)	0.5308
Suspicion of/secured	3 (14.3%)	2 (25.0%)	1 (7.7%)	0.5308
**Post-Traumatic Stress Disorder (IES-R)**				
IES-R score positive for PTSD (>0)	6 (28.6%)	4 (50.0%)	2 (15.4%)	0.1462
IES-R sum score	−1.3 (−4.2–2.6)	−0.6 (−4.1–1.7)	−1.7 (−4.2–2.6)	0.1668
IES-R intrusion	14.5 (0–27)	17.0 (1–26)	12.9 (0–27)	0.4021
IES-R avoidance	15.0 (0–36)	17.6 (0–30)	13.4 (3–36)	0.2923
IES-R hyperarousal	15.6 (0–33)	19.4 (2–31)	13.2 (0–33)	0.1555

## Data Availability

The datasets presented in this article are not readily available because the data are part of an ongoing study (FEOS II).

## Supplementary Materials

The following supporting information can be downloaded at https://www.mdpi.com/article/10.3390/jcm14072206/s1, Figure S1: A: bullseye plot; Table S1: Comparison of quality of life of FEOS with DEGS1, Table S2: Influencing factors of PTSD: Comparison of patients with and without PTSD.
