# Supplementary material for: Quality of Life and Mental Health in COVID-ARDS Survivors After V-V ECMO Support: Results from the Freiburg ECMO Outcome Study (FEOS)
Source: J Clin Med. 2025 Mar 24;14(7):2206. doi: 10.3390/jcm14072206 (PMC11989409; doi:10.3390/jcm14072206)
Supplement: Supplementary file 1 [file jcm-14-02206-s001.zip › jcm-3520032-supplementary.pdf]

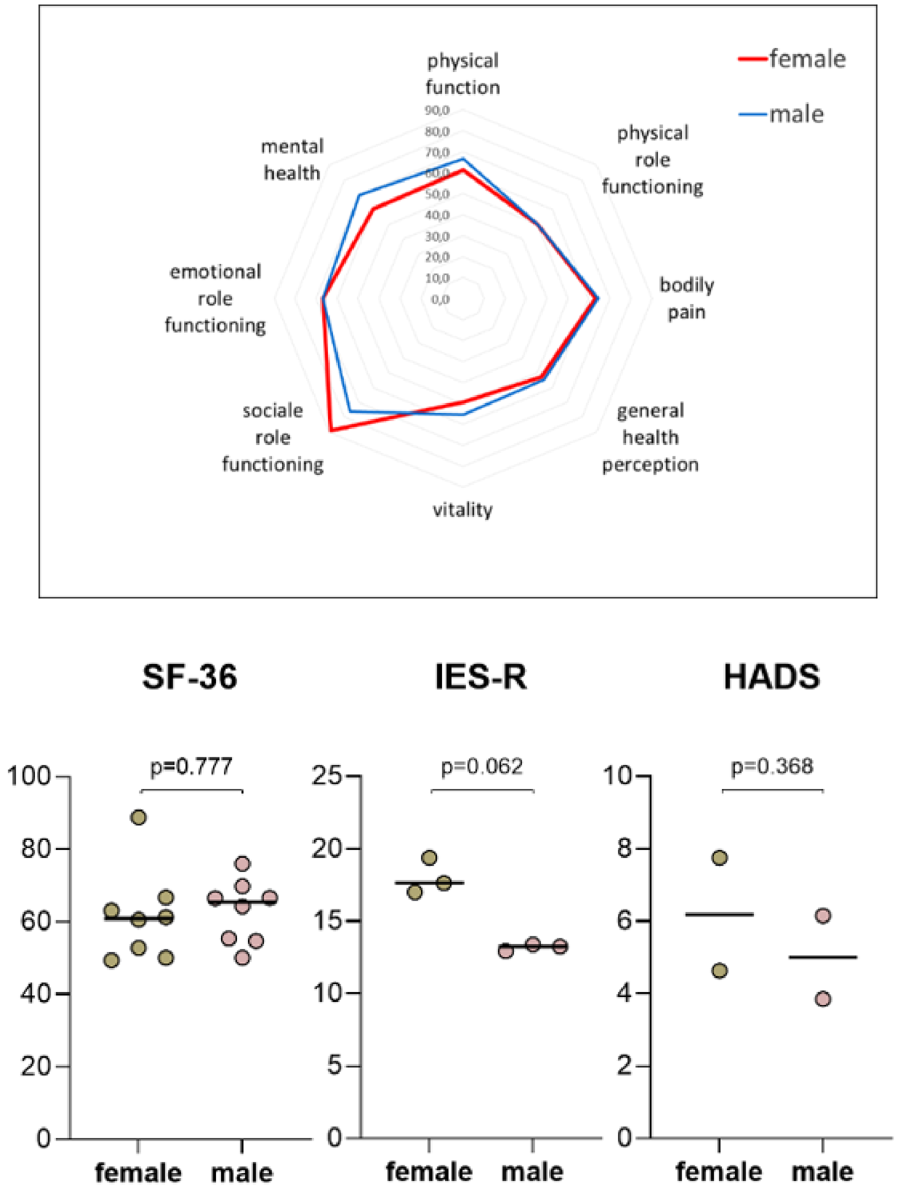

Figure S1. A: bullseye plot: Health-related quality of life as estimated by the SF-36 score in FEOS (comparison male and female patients). Higher scores denote better health-related quality of life. B: Dot plot of SF-36, IE-S and HADS in male and female patients.

Table S1. Comparison of quality of life of FEOS with DEGS1 (Significance calculated by: [a] ttest, [b] 2way ANOVA).

FS-36	FEOS			DEGS1			p-value	
	mean	std	n	mean	std	n	ttest	anova
Physical functioning	64.5	26.8	21	84.5	35.8	7688	**0.0106** [a]	<0.0001 [b]
Physical role functioning	50.0	40.3	21	82.1	33.5	7667	**<0.0001** [a]	
Bodily pain	63.9	29.3	21	74.8	33.8	7784	0.1379 [a]	
General health perceptions	54.0	20.8	21	69.3	26.9	7708	**0.0089** [a]	
Vitality	53.1	22.1	21	61.6	24.7	7729	0.1146 [a]	
Social role functioning	80.8	24.7	21	86.1	29.3	7795	0.4103 [a]	
Emotional role functioning	66.6	39.5	21	86.0	29.0	7662	**0.0023** [a]	
Mental health	66.3	21.9	21	72.9	22.4	7719	0.1769 [a]	

Table S2. Influencing factors of PTSD: Comparison of patients with and without PTSD (Significance calculated by: [a] ttest, [b] 2way ANOVA).

**Influencing factors for PTSD**	All (n=21)	PTSD (n=6)	No PTSD (n=15)	
Age (years)	51.21 ± 11.44	39.01 ± 8.28	56.07 ± 8.62	**0.0006**
Female sex	8 (38.1%)	4 (66.6%)	4 (26.6%)	0.1462
Length of Hospital Stay (days +/- SD)	62.95 ± 39.76	49.33 ± 24.90	68.79 ± 30.56	0.1834
Length of ICU Stay (days +/- SD)	51.43 ± 25.46	40.33 ± 19.77	55.87 ± 26.69	0.2146
Duration of invasiv Ventilation (days)	47.24 ± 24.18	36.50 ± 19.99	51.53 ± 24.97	0.2060
Duration of ECMO (days)	29.06 ± 20.42	24.99 ± 16.00	30.69 ± 22.23	0.5766
Blood gas analysis before ECMO-Implantation:				
• pO_2 (mmHg)	59.69 ± 14.33	48.83 ± 10.32	65.41 ± 17.79	**0.0471**
• SaO_2 (%)	83.56 ± 11.94	77.70 ± 18.82	86.07 ± 7.04	0.1446
• pCO_2(mmHg)	64.82 ± 26.43	50.22 ± 9.98	71.08 ± 29.02	0.1058
Ventilator settings and measurements before ECMO-Implantations:				
• P/F-ratio before ECMO-Implantation	64.15 ± 20.12	59.37 ± 28.89	66.06 ± 16.32	0.5053
• Tidalvolume (ml)	418 ± 193	373 ± 174	438 ± 203	0.5002
• Compliance (ml/	30.57 ± 27.87	21.52 ± 13.25	34.45 ± 31.84	0.3538
